# Scalable fabrication of gas sensors via spark-ablation printing of semiconductive metal oxide nanoparticles and heterostructures

**DOI:** 10.1038/s41378-026-01208-1

**Published:** 2026-04-21

**Authors:** Wenke Fu, Zhenyuan Tang, Yanshu Gu, Xiaoli Shao, Jingyuan Zhang, Ziying Hu, Mingdi Zhang, Jixiang Li, Zeming Jin, Xia Liu, Min Tu

**Affiliations:** 1 School of Integrated Circuits and Electronics, MIIT Key Laboratory for Low-Dimensional Quantum Structure and Devices, Beijing Institute of Technology, Beijing, 100081 China; 2 State Key Laboratory of Transducer Technology, Shanghai Institute of Microsystem and Information Technology, Chinese Academy of Sciences, Shanghai, 200050 China; 3 Center of Materials Science and Optoelectronics Engineering, University of Chinese Academy of Sciences, Beijing, 100049 China; 4 2020 X-Lab, Shanghai Institute of Microsystem and Information Technology, Chinese Academy of Sciences, Shanghai, 200050 China; 5 School of Graduate Study, University of Chinese Academy of Sciences, Beijing, 100049 China

**Keywords:** Materials science, Engineering

## Abstract

Semiconductive metal oxide (SMO) gas sensors are extensively used in air monitoring, industrial safety, and hazardous-gas detection due to their high sensitivity, low cost and low power consumption. Advances in nanotechnology have enabled precise control over the morphology and electronic structure of SMOs, thereby enhancing their sensing performances. However, the implementation of nanoscale SMOs into gas sensors typically involves two steps consisting of material synthesis and subsequent transfer onto device substrates, which face challenges in ensuring high uniformity and reproducibility. Here, we report a scalable gas sensor fabrication strategy based on spark-ablation printing, which enables the simultaneous synthesis and direct deposition of SMO films onto micro hotplate sensor chips. This one-step process allows the integration of both pristine and noble-metal-decorated SMOs, achieving ppb-level gas detection limit with excellent device-to-device consistency. Furthermore, sensor arrays composed of diverse SMO materials, when integrated with machine-learning algorithms, enable the accurate classification of four gases (>99%), demonstrating the potential of this approach for scalable and intelligent gas-sensing applications.

**Figure:**
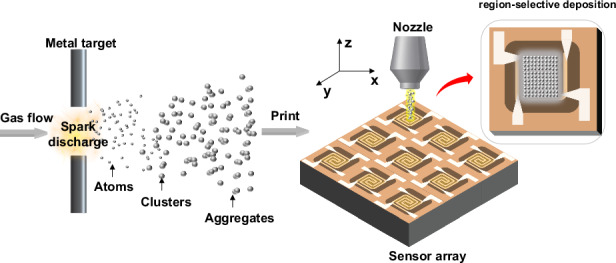
Spark-ablation printing employs pulsed high-voltage discharges to vaporize electrode materials, generating nanoparticles that are transported by the gas flow and deposited onto MEMS microheater arrays as porous sensing films. This solvent-free and region-selective deposition technique enables scalable integration of diverse metal oxides, providing a robust platform for high-performance gas sensing

Spark-ablation printing employs pulsed high-voltage discharges to vaporize electrode materials, generating nanoparticles that are transported by the gas flow and deposited onto MEMS microheater arrays as porous sensing films. This solvent-free and region-selective deposition technique enables scalable integration of diverse metal oxides, providing a robust platform for high-performance gas sensing

## Introduction

Gas sensors are indispensable for environmental monitoring^1–3^, industrial safety^4,5^, and public health protection^6–8^. The rapid evolution of intelligent sensing technologies has created a pressing need for miniaturized, energy-efficient, and highly integrated platforms capable of real-time and low-power gas detection^9–12^. Single sensors often exhibit limited selectivity, thereby restricting their applicability in complex gas backgrounds^13,14^. To overcome this drawback, Micro-Electro-Mechanical Systems (MEMS)-based sensor arrays incorporating multiple metal-oxide materials have been developed and combined with machine learning algorithms to leverage multidimensional response patterns for reliable gas identification and accurate concentration estimation^15–19^. The effectiveness of such arrays critically depends on device-to-device consistency, as demonstrated by Park et al.^20^, who showed that array uniformity is a prerequisite for reliable machine learning-enabled gas classification in practical electronic-nose systems. The fabrication of multi-material sensor arrays remains constrained by the challenge of achieving consistent, region-selective deposition of sensitive materials, which is essential for uniform performance and scalable production. Conventional wet-chemical routes, such as hydrothermal, sol-gel, and template-assisted methods^21,22^, can produce high-performance nanomaterials but generally lack the spatial selectivity and reproducibility required for wafer-level integration. In contrast, vapor-phase techniques including physical vapor deposition (PVD)^23–25^ and chemical vapor deposition (CVD)^26–28^ provide better control over thickness and composition but typically yield dense films with low porosity and surface area, which weakens gas-solid interactions and limits sensitivity. Accordingly, a straightforward and region-selective deposition strategy that enables the formation of porous and uniform metal oxide films on sensor platforms is critically needed to advance next-generation gas sensor arrays.

**Fig. 1:**
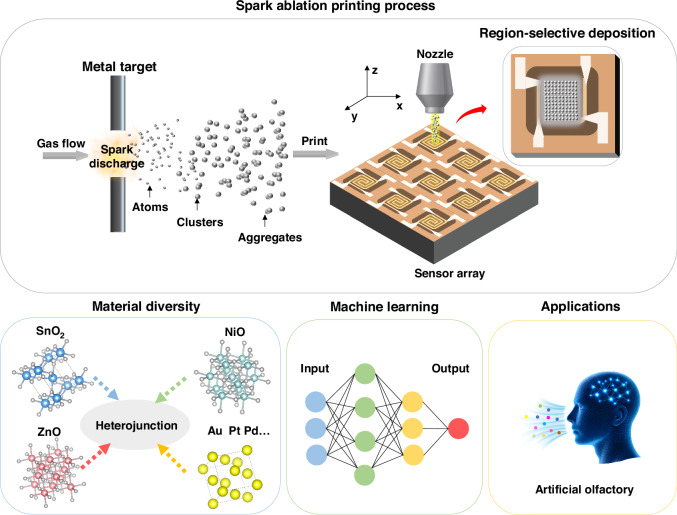
Schematic illustration of the spark-ablation printing approach for fabricating gas sensor arrays. Spark ablation uses pulsed high-voltage discharges between metal electrodes to vaporize the target. The resulting vapor species are rapidly quenched and diluted in the gas flow, where collisions induce their growth into nanoparticles that merge into nanoporous aggregates. These aerosol particles are then carried by the gas flow and deposited in a spatially controlled manner onto MEMS microheater substrates. This method is compatible with various SMOs and noble-metal-decorated SMO heterojunctions. Integrated with machine-learning algorithms, the resulting arrays enable reliable multi-gas recognition for artificial-olfaction applications

In this work, we present a spark-ablation printing strategy that enables the simultaneous generation and deposition of metal-oxide nanoparticles onto sensor platforms (Fig. 1). This solvent-free and transfer-free process affords precise positional control and facilitates the fabrication of high-performance gas sensors. Spark ablation employs high-voltage discharges between conductive electrodes to vaporize the target material^29–32^. The vaporized atomic and cluster species generated by each spark are rapidly quenched and diluted in the gas flow. Subsequent collisions between atoms and clusters lead to immediate sticking and growth into nanoparticles, which may further coalesce into nanoporous aggregates. These aerosol particles are subsequently transported by the gas flow and deposited in a spatially controlled manner onto MEMS microheater substrates. This solvent-free process yields porous metal-oxide films with high chemical purity. When combined with spatially programmed deposition, it enables region-selective patterning with high uniformity across sensor arrays. Using this strategy, MEMS gas sensors based on SnO_2_, ZnO, NiO, and Au-functionalized SnO_2_, were fabricated, showing ppb-level detection limit and high device-to-device consistency. Moreover, multi-material sensor arrays integrated with machine-learning algorithms enabled accurate discrimination of multiple gases, highlighting the promise of spark-ablation printing for portable gas sensing and artificial olfaction.

## Results and discussion

**Fig. 2:**
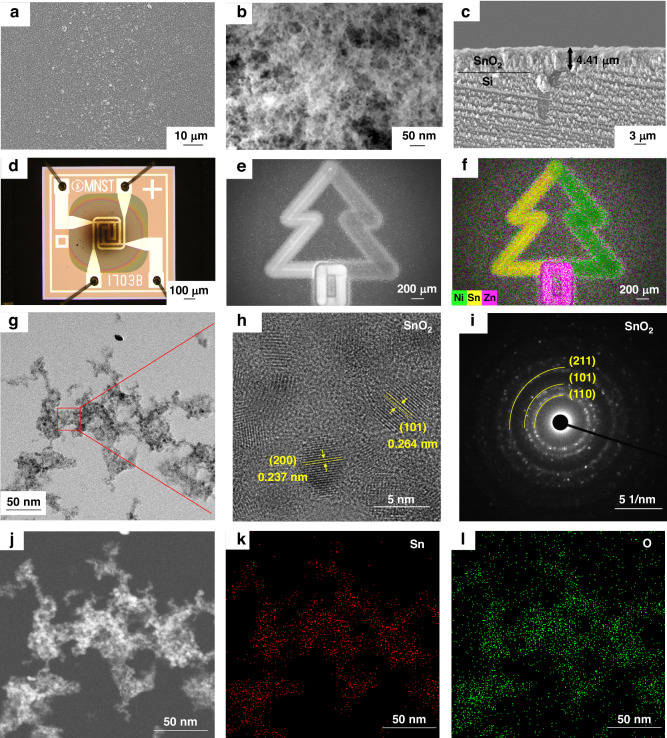
Characterization of SMO films deposited by spark ablation printing. **a**, **b** SEM images of the spark-ablated SnO_2_ film. **c** Cross-sectional SEM image of the SnO_2_ film. **d** Image of a SnO_2_ film region-selectively deposited on a MEMS microheater substrate. **e**, **f** SEM image and corresponding EDS mapping of a multicomponent “Christmas tree” pattern composed of SnO_2_, ZnO, and NiO lines. **g** TEM image of SnO_2_ nanoparticles produced by spark ablation. **h** HRTEM image revealing lattice fringes corresponding to the (200) and (101) planes of tetragonal SnO_2_. **i** SAED pattern indexed to the (110), (101), and (211) planes of the SnO_2_ nanoparticles. HAADF-STEM image of the SnO_2_ nanoparticles shown in (**j**) and the corresponding EDS elemental maps of Sn (**k**) and O (**l**)

We first utilize spark-ablation printing to fabricate a prototype SnO_2_ film, a representative gas-sensing material widely employed in chemiresistive gas sensors. In a purely inert Ar atmosphere, spark ablation generates metallic nanoparticles from the electrodes. When a small amount of oxygen is introduced (0.1% O_2_/Ar), these highly reactive particles undergo in-flight oxidation, resulting in the direct formation and deposition of nanocrystalline SnO_2_ films^33^. Scanning Electron Microscopy (SEM) revealed that the deposited SnO_2_ films consist of loosely packed nanoparticles forming porous networks (Fig. 2a, b). This porous morphology provides abundant active sites and facilitates rapid gas diffusion, thereby enhancing sensing performances^34–36^. The film thickness could be controlled by adjusting the printing speed: higher speeds produced thinner films, whereas lower speeds led to thicker layers, while the porous morphology was well preserved. (Figs. S1, S2). Cross-sectional SEM image confirms the uniform thickness of the deposited films (Fig. 2c). The direct-writing capability of spark-ablation printing enables regioselective deposition, as demonstrated by patterned SnO_2_ film within a 400 µm × 400 µm sensing area (Fig. 2d). Furthermore, sequential deposition of different oxides on a single chip produced multicomponent patterns, exemplified by a ‘Christmas tree’ pattern composed of spatially distributed SnO_2_, ZnO, and NiO lines (Fig. 2e, f), highlighting the versatility of spark-ablation printing for region-selective integration of multiple materials.

Transmission electron microscopy (TEM) revealed that the SnO_2_ nanoparticles generated by spark ablation were loosely assembled into porous aggregates (Fig. 2g). High-resolution TEM (HRTEM) resolved lattice fringes of 0.237 nm and 0.264 nm, corresponding to the (200) and (101) planes of tetragonal SnO_2_, respectively (Fig. 2h). The selected-area electron diffraction (SAED) pattern displayed continuous rings, confirming their polycrystalline nature without detectable secondary phases (Fig. 2i). Energy-dispersive X-ray spectroscopy (EDS) mapping further verified the homogeneous spatial distribution of Sn and O within the aggregates (Fig. 2j–l). Collectively, these results demonstrate that spark-ablated SnO_2_ exhibits high crystallinity, compositional uniformity, and porous morphology, providing abundant active sites and facilitating efficient gas diffusion, thereby establishing a robust structural basis for high gas-sensing performance.

**Fig. 3:**
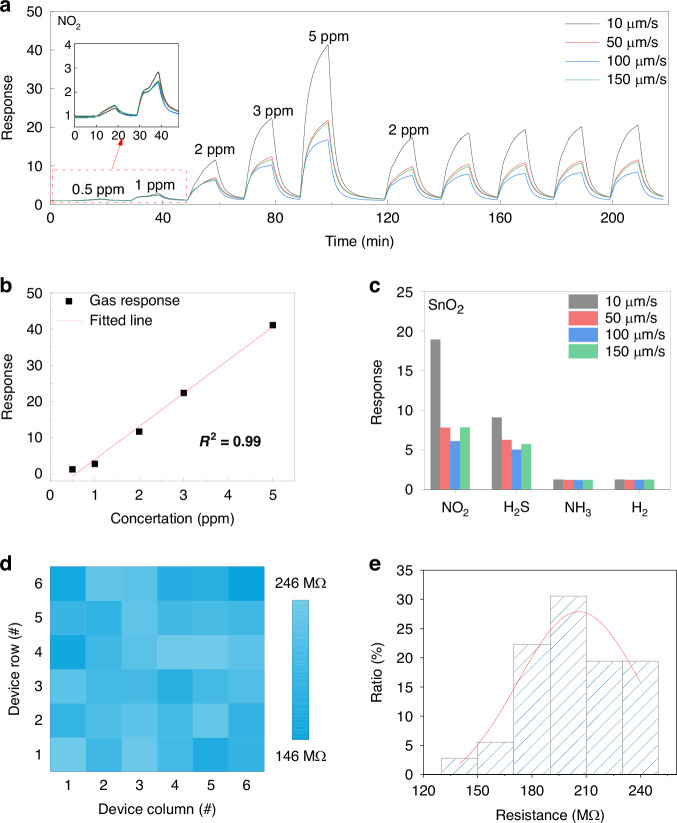
Gas sensing performances fabricated SnO2 sensors. **a** Dynamic responses of a SnO_2_ gas sensor to NO_2_ concentrations of 0.5, 1, 2, 3, and 5 ppm at 325 °C, including five repeated exposure cycles at 2 ppm. **b** Concentration-dependent responses of a SnO_2_ gas sensor printed at 10 μm/s, showing a nearly linear relationship with NO_2_ concentration (*R*^2^ = 0.99). **c** Sensing selectivity of a SnO_2_ gas sensor toward NO_2_, H_2_S, NH_3_, and H_2_ (2 ppm each) at 325 °C. **d** Resistance mapping of a 6 × 6 SnO_2_ sensor array (bias voltage 1 V; operating temperature 300 °C) fabricated by spark ablation printing, exhibiting high uniformity with a coefficient of variation (CV) of 0.129. **e** Histogram of baseline resistance values from the sensor array shown in (**d**) evidencing the uniform distribution of device resistances

By regioselective printing of the porous SnO_2_ layer on the sensing area of a MEMS micro hotplate, a chemiresistive gas sensor was formed. Nitrogen dioxide (NO_2_) is not only a toxic pollutant but also a key precursor of O_3_ and PM_2.5_, with chronic exposure linked to asthma, lung disease, and increased mortality even at ppb levels^28,37,38^. These hazards highlight the imperative for highly sensitive and rapid NO_2_ detection. At the optimal operating temperature of 325 °C (Fig. S3), the SnO_2_ sensors exhibited reversible responses toward NO_2_ in the concentration range of 0.5–5 ppm (Fig. 3a). Upon repeated exposure to 2 ppm NO_2_, the response signals remained nearly identical, confirming the reproducible performance of the sensors. Furthermore, the response to NO_2_ exhibited an almost linear dependence on concentration, yielding an *R*^2^ value of 0.99 (Fig. 3b), which confirms reliable quantitative detection and facilitates calibration for practical use. Selectivity was subsequently assessed, as it represents a critical parameter in gas sensing. SnO_2_ films printed at different speeds were tested against 2 ppm of NO_2_, H_2_S, NH_3_, and H_2_ (Fig. 3c and Fig. S4). The response to NO_2_ was substantially higher than to the other tested gases, confirming the selective nature of the sensors. In addition, films printed at slower speeds exhibited stronger responses to NO_2_, which can be attributed to the formation of relatively thicker and more continuous porous networks that provide a larger number of active sites. Apart from the performance of sensitivity and selectivity, long-term stability is another essential for practical application. Therefore, we tested a SnO_2_ sensor after storage under ambient conditions for 1 month. The aged device was then operated continuously at 325 °C for 8 h and subjected to three measurement phases of 2 ppm NO_2_ cycling, with five exposure–recovery cycles in each phase (Fig. S5a). The response transients remained almost unchanged throughout the test, and the average response to 2 ppm NO_2_ after aging (derived from the three phases) was nearly identical to that of the pristine device (Fig. S5b), with only minor variations within the experimental error. These observations indicate negligible drift or degradation, confirming that the spark-ablated SnO_2_ films maintain robust long-term stability under the tested conditions.

Consistency among sensor devices is a critical prerequisite for scalable integration. To evaluate the potential of spark-ablation printing for large-scale fabrication, SnO_2_ films were deposited onto a 6 × 6 sensor array within 8 min. I–V measurements in air at 300 °C (Fig. 3d) revealed an average resistance of 204.7 ± 26.6 MΩ with a coefficient of variation (CV) of 0.129. Statistical analysis further confirmed the high film uniformity across the array (Fig. 3e). Collectively, these results demonstrate that spark-ablation printing enables the fabrication of SnO_2_ gas sensors with high device-to-device uniformity, underscoring its strong potential for scalable array integration.

**Fig. 4:**
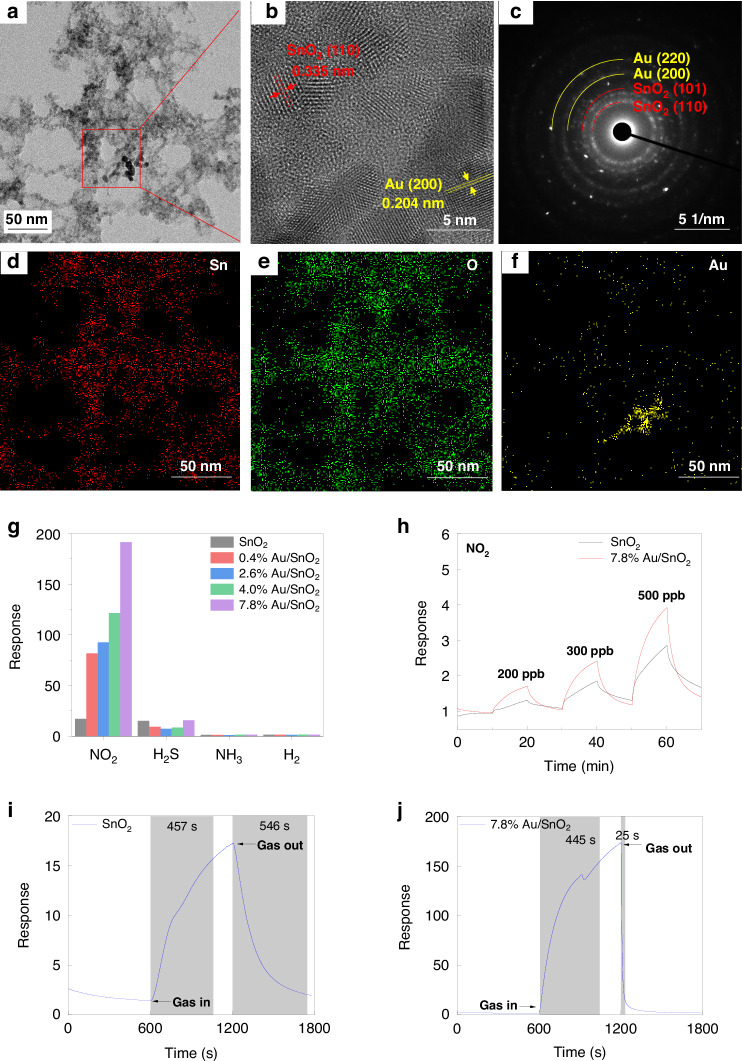
Characterization of Au/SnO2 nanoparticles and sensing performances of Au/SnO2-based gas sensors. **a** TEM image of Au/SnO_2_ nanoparticles with uniformly dispersed Au decorations. **b** HRTEM image showing lattice fringes of SnO_2_ (110) and Au (200). **c** SAED pattern indexed to tetragonal SnO_2_ (110, 101) and fcc Au (200, 220), confirming the coexistence of both phases. **d**–**f** EDS elemental maps of Sn, O, and Au, evidencing uniform elemental distribution. **g** Dynamic responses of sensors with different Au loadings (0–7.8 wt%) to 2 ppm target gases, illustrating sensitivity enhancement by Au incorporation. **h** Responses of SnO_2_ and 7.8 wt% Au/SnO_2_ sensors to low-concentration (200–500 ppb) NO_2_. **i**, **j** Response and recovery curves of SnO_2_ and 7.8 wt% Au/SnO_2_ sensors toward 2 ppm NO_2_

With device uniformity and scalability demonstrated, gas sensing performance enhancement was subsequently investigated through noble metal functionalization. Noble metals such as Au, Pt, and Pd possess high work functions and strong electron-trapping abilities, facilitating charge transfer at the SMO interface^39–41^. Together with their intrinsic catalytic activity, these properties can enhance sensor sensitivity, lower operating temperature, and improve selectivity, making noble metals widely adopted for functionalization in SMO-based sensors. Beyond SMOs, spark ablation can also be employed for the deposition of noble-metal clusters, which can be controllably integrated into SMO films. On this basis, SnO_2_ films with varying Au contents were fabricated via co-ablation of Au and Sn electrodes (Fig. S6). In this process, the discharge voltage and current define the spark energy and frequency, which determine the ablated mass from each electrode and thus allow precise regulation of Au amount. TEM and HRTEM analyses revealed aggregated SnO_2_ nanoparticles decorated with Au, as indicated by lattice fringes corresponding to Au (200) and SnO_2_ (110) (Fig. 4a, b). The SAED pattern further confirmed the coexistence of tetragonal SnO_2_ and face-centered cubic (fcc) Au (Fig. 4c). Elemental mapping showed homogeneous distributions of Sn and O along with distinct Au signals, validating the effective incorporation of Au into SnO_2_ (Fig. 4d–f).

The effect of Au incorporation on the sensing characteristics of SnO_2_ sensors was evaluated at the optimal operating temperature of 275 °C (Fig. S7). The average response to 2 ppm NO_2_ increased significantly with Au content, with the 7.8 wt% Au/SnO_2_ sensor showing an approximately 11-fold enhancement over pristine SnO_2_ sensor while maintaining negligible responses to H_2_S, NH_3_, and H_2_ (Fig. 4g and Fig. S8). At low NO_2_ concentrations (200–500 ppb), the 7.8 wt% Au/SnO_2_ sensor exhibited markedly higher responses than the sensor based on pristine SnO_2_ (Fig. 4h). The limits of detection (LOD) were determined to be 1 ppb for SnO_2_ and 0.11 ppb for 7.8 wt% Au/SnO_2_ sensors (Fig. S9), respectively, underscoring the substantial sensitivity enhancement conferred by Au incorporation. Moreover, the 7.8 wt% Au/SnO_2_ sensor displayed a recovery time of 25 s at 2 ppm NO_2_, compared to 546 s for pristine SnO_2_ (Fig. 4i, j), demonstrating significantly accelerated recovery speed. The electronic and catalytic functions of Au, facilitate charge transfer at the Au–SnO_2_ interface^42–45^ and promote oxygen activation^46–48^ thereby accounting for the enhanced response, selectivity, lower detection limit, and faster recovery (Fig. S10).

**Fig. 5:**
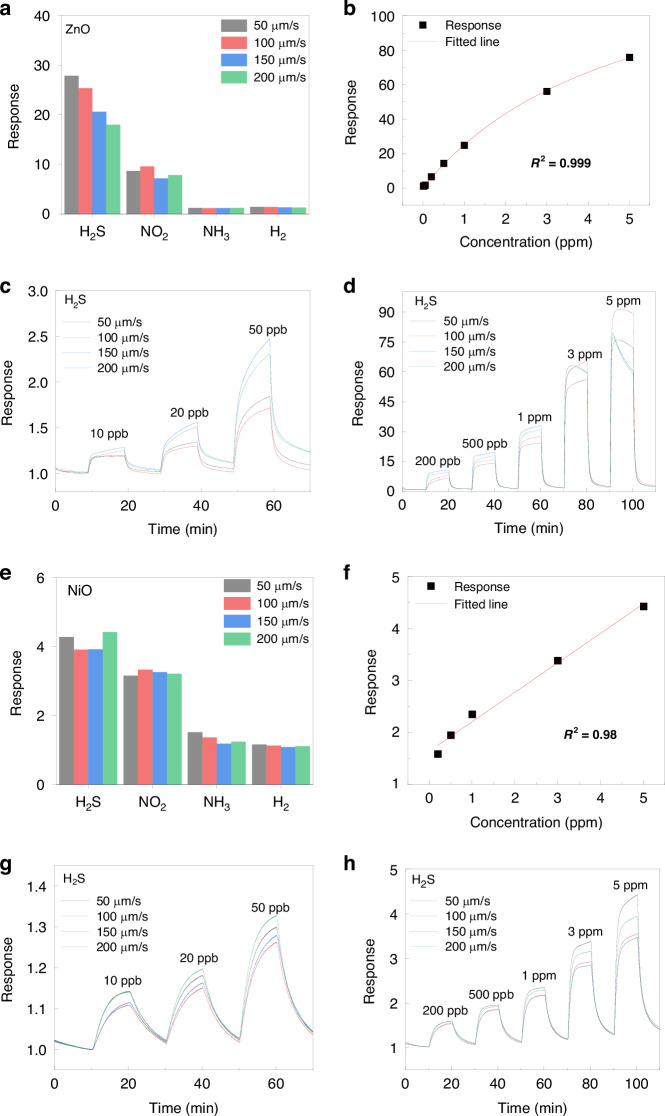
Gas sensing performances of spark ablation printed ZnO and NiO sensors. **a** Responses of the ZnO sensor toward different target gases (2 ppm) at 475 °C; **b** H_2_S response–concentration curve of a ZnO sensor printed at 50 μm/s, fitted with the Langmuir adsorption model (*R*^2^ = 0.999); **c**, **d** dynamic responses of ZnO sensors with different printing speed to H_2_S at 10–50 ppb and 200 ppb-5 ppm, respectively; **e** responses of the NiO sensor toward different target gases (5 ppm) at 225 °C; **f** H_2_S response-concentration curve of a NiO sensor printed at 50 μm/s, fitted with the linear model (*R*^2^ = 0.98); **g**, **h** dynamic responses of NiO with different printing speed to H_2_S at 10–50 ppb and 200 ppb-5 ppm, respectively

To demonstrate the versatility of spark-ablation printing, gas sensors were fabricated from two representative SMOs: ZnO (n-type) and NiO (p-type)^49–54^. Hydrogen sulfide (H_2_S) is a highly toxic gas with a characteristic rotten-egg odor, can cause severe respiratory irritation and fatal poisoning at high concentrations, while long-term exposure to low levels is associated with neurological and respiratory disorders^55,56^. These risks underscore the need for accurate, sensitive, and rapid detection of H_2_S. Both ZnO and NiO gas sensors show high responses towards low-concentration H_2_S with high repeatability. Their sensing selectivity and repeatability were assessed under repeated exposures to H_2_S, NO_2_, NH_3_, and H_2_ at their respective optimal operating conditions and at printing speeds of 50-200 μm/s (Figs. 5a, e and S11–S14). Their sensing behavior was further examined across a wide concentration range, from 10 to 50 ppb up to 5 ppm (Fig. 5c, d, g, h). Under these conditions, both sensors exhibited distinct concentration-dependent responses, with the ZnO sensor following a Langmuir adsorption model (*R*^2^ = 0.999; Fig. 5b) and the NiO sensor displaying a strong linear dependence (*R*^2^ = 0.98; Fig. 5f). Stable outputs were maintained down to 10 ppb, corresponding to LOD of 0.17 ppb for ZnO and 2.5 ppb for NiO, respectively. Also, both sensors showed fast responses towards 5 ppm H_2_S, with response times of 43 s for ZnO and 99 s for NiO, respectively. Compared with previously reported SMO-based gas sensors, the devices fabricated via spark-ablation printing exhibit top-tier performance in terms of response, LOD, and response time (Table S2). Moreover, this approach demonstrates strong potential for scalable batch fabrication, further highlighting its suitability for high-performance gas sensor production.

**Fig. 6:**
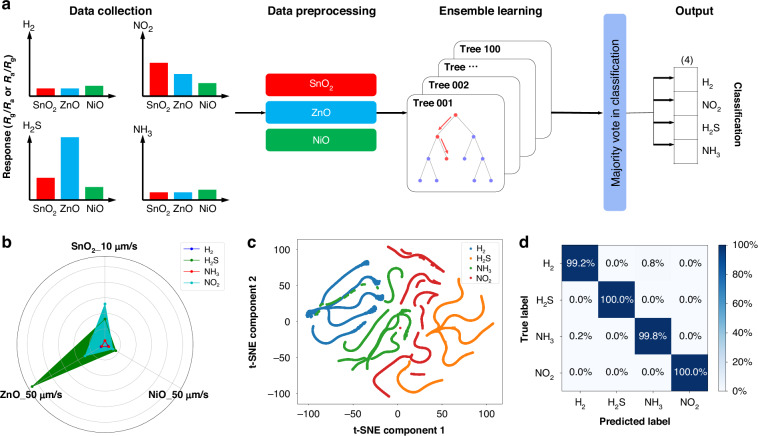
Machine learning results for gas identification using the fabricated sensor array. **a** Flowchart of the random forest framework used for four-gas classification; **b** radar chart visualization of the sensing dataset showing the distinctive response patterns of SnO_2_, ZnO, and NiO sensors; **c** two-dimensional t-distributed stochastic neighbor embedding (t-SNE) plot of the dataset colored by gas type, illustrating the clustering of gas responses; **d** confusion matrix of predicted gas types obtained from the random forest classifier, demonstrating nearly perfect classification performance across all four gases

The material versatility of spark-ablation printing enables the fabrication of multi-oxide sensor arrays that exploit complementary response patterns to achieve reliable discrimination of multiple gases^57–59^. Accordingly, machine learning was applied to classify gases based on the responses of SnO_2_, ZnO, and NiO sensors (Fig. 6a). Feature distributions were first visualized using radar plots (Fig. 6b), and t-distributed stochastic neighbor embedding (t-SNE) was employed to project the high-dimensional data into two dimensions (Fig. 6c). Distinct gas clusters were observed, though partial overlap between H_2_ and NH_3_ reflected the limitations of unsupervised visualization. To overcome this, a random forest (RF) classifier was implemented within an ensemble learning framework. By aggregating multiple decision trees trained on bootstrap samples and random feature subsets, RF reduces overfitting and improves robustness. Confusion matrix analysis confirmed nearly perfect classification across all four gases, with only minor (<1%) misclassifications between H_2_ and NH_3_ (Fig. 6d). To place these results in a broader context, the gas classification performance is compared with recent ML-assisted studies (Table S3), showing that our approach achieves competitive accuracy. These results underscore the synergistic potential of spark-ablation-fabricated oxide sensor arrays and machine learning, providing a robust pathway toward intelligent gas sensing in environmental monitoring and safety.

## Conclusion

In conclusion, we demonstrated the effectiveness of the spark-ablation printing approach for fabricating high-performance MEMS gas sensors. This one-step approach allows the simultaneous generation and direct deposition of nanosized SMOs and noble-metal-decorated SMOs onto the sensing regions, effectively overcoming the limitations of the conventional synthesis-transfer workflows. The fabricated sensors show high sensing performances towards H_2_S and NO_2_ gases, along with excellent uniformity across a large sensor array. Leveraging the versatility this technique, multi-material sensor arrays were further fabricated and integrated them with machine learning, achieving near-perfect classification of NO_2_, H_2_S, NH_3_, and H_2_ with misclassification rates below 1%. Overall, the developed spark-ablation workflow unifies material synthesis and device integration in a single and scalable process, providing a versatile route toward multi-oxide electronic-nose platforms for next-generation environmental, healthcare, and industrial sensing applications.

## Experimental section

### Materials

Tin (Sn, 99.99%), zinc (Zn, 99.99%), nickel (Ni, 99.99%), and gold (Au, 99.99%) electrodes were purchased from VSParticle (Netherlands). Air, O_2_/Ar, NO_2_, H_2_, H_2_S, and NH_3_ gases used in the experiments were purchased by Shanghai Weichuang Standard Gas Analytical Technology.

### Spark ablation printing

Porous metal oxide thin films were fabricated using a spark ablation system (VSP G1, VSParticle, Netherlands). Short high-voltage discharges (<10 μs) between metal electrodes generated transient plasmas that ablated the electrode material. The vaporized species were rapidly quenched and diluted in an O₂/Ar flow, leading to condensation and growth into 1–20 nm nanoparticles. These nanoparticles further agglomerated into fractal aggregates and were carried by the gas flow through a nozzle for spatially controlled deposition onto MEMS microheater substrates, forming porous thin films.

### Spark ablation printing of metal oxide films

SnO_2_, ZnO, and NiO films were deposited by oxidizing Sn, Zn, and Ni electrodes in a 0.1% O_2_/Ar atmosphere using spark ablation. The total gas flow was maintained at 1 L min⁻¹. Spark discharges were generated by charging a 30 nF storage capacitor to 1.3 kV and discharging at an average current of 8 mA, corresponding to a discharge repetition rate of approximately 205 Hz (estimated from $f=I/(C{V}_{d})$). The nozzle was positioned at an approximate distance of 800 μm above the microheater surface, which resulted in a deposited film covering an area of about 9.4 × 10^4^ μm^2^ on the chip. The generated oxide nanoparticles were transported by the gas flow and deposited onto substrates, forming uniform porous films. Au-loaded SnO_2_ films were obtained by simultaneous spark ablation of Sn and Au electrodes. The SnO_2_ electrode was maintained at 1.3 kV and 8 mA, the voltage and current applied to the Au electrode were regulated within the ranges of 0.2–0.4 kV and 0.1–1.1 mA, respectively, to control the Au loading (detailed parameters for each sample are listed in Table S1).

### Gas sensor fabrication

Gas sensors were fabricated by spark ablation printing of pristine and noble metal–modified oxides onto MEMS microheaters. (SA-MHP-3.8, Beijing Sinoagg, China). Each device comprised a silicon substrate with integrated platinum heating and sensing electrodes. The localized heating zone (0.4 mm × 0.4 mm) provided precise thermal control. The entire chips (3.8 mm × 3.8 mm) were ceramic-packaged with gold wire connections. The integrated heater exhibited a resistance of approximately 150 Ω and could reach temperatures up to 550 °C at a power input of 65 mW.

### Measurement and characterization

Field-emission scanning electron microscopy (FE-SEM, Zeiss Gemini 300, Germany; 5 kV) was employed to examine the surface morphology of the sensing films, and elemental compositions were analyzed by energy-dispersive X-ray spectroscopy (EDS). High-resolution transmission electron microscopy (HR-TEM, JEM-F200, JEOL, Japan) combined with selected-area electron diffraction (SAED) and STEM-EDS mapping was used to characterize the crystallographic structures, nanoparticle interfaces, and elemental distributions. Film thickness was measured using a stylus profilometer (Chotest CP200, China). Electrical measurements were conducted using a probe station connected to a source measurement unit (SMU, Keysight B2901BL, USA). Current–voltage (I–V) characteristics were recorded from −1 to +1 V with a step of 0.02 V, and resistance values were extracted at 300 °C.

### Gas sensing measurement

Gas-sensing performance was evaluated using a CGS-8R system equipped with a humidity-controlled gas delivery module (DGL Series, Beijing Sinoagg, China). The heating plate with deposited films was mounted in a sealed chamber, and a controlled current was applied to reach the desired operating temperature. Test gases (e.g., NO_2_, H_2_S) were introduced by alternating the flow between dry air and target gases, with concentrations precisely adjusted by mixing ratios and total flow. All gas-sensing measurements were performed in dry air (H_2_O < 5 ppm) as the carrier and background atmosphere. Device resistance was continuously monitored, and the sensor response (S) was defined as S = Rg/Ra for oxidizing gases and S = Ra/Rg for reducing gases, where Ra and Rg denote the resistances in air and in the target gas, respectively.

### LOD calculations

The limit of detection (LOD) was calculated based on the standard deviation of the response (Sᵧ) and the slope of the calibration curve (S). The LOD was estimated according to the equation LOD = 3 × (Sᵧ/S)^60^. An illustrative example of the calibration curve and baseline noise used to determine S and Sᵧ for the 7.8 wt% Au/SnO_2_ sensor is provided (Fig. S9).

### Data processing

Response data from SnO_2_, ZnO, and NiO sensors toward H_2_, NO_2_, H_2_S, and NH_3_ were collected to construct the classification dataset. The dataset was randomly partitioned into training (50%), validation (20%), and testing (30%) subsets for model training, hyperparameter tuning, and performance evaluation, respectively.

## Supplementary information

Supporting Information

## Data Availability

The data that support the findings of this study are available from the corresponding author upon reasonable request.
