# Supplementary material for: Scalable fabrication of gas sensors via spark-ablation printing of semiconductive metal oxide nanoparticles and heterostructures
Source: Microsyst Nanoeng. 2026 Apr 21;12:141. doi: 10.1038/s41378-026-01208-1 (PMC13100202; doi:10.1038/s41378-026-01208-1)
Supplement: Supplementary file 1 — Supporting Information [file 41378_2026_1208_MOESM1_ESM.docx]

Supporting Information

**Scalable Fabrication of Gas Sensors** **via Spark-Ablation Printing of Semiconductive Metal Oxide Nanoparticles and Heterostructures**

Wenke Fu,^1,2+^ Zhenyuan Tang,^2,3,4,5+^ Yanshu Gu, ^2,3,4,5^ Xiaoli Shao,^2^ Jingyuan Zhang,^2^ Ziying Hu,^1^ Mingdi Zhang,^1^ Jixiang Li,^1^ Zeming Jin,^1^ Xia Liu^1*^ and Min Tu^2,3,4,5*^

^1^School of Integrated Circuits and Electronics, MIIT Key Laboratory for Low-Dimensional Quantum Structure and Devices, Beijing Institute of Technology, Beijing 100081, China

^2^State Key Laboratory of Transducer Technology, Shanghai Institute of Microsystem and Information Technology, Chinese Academy of Sciences, Shanghai 200050, China.

^3^Center of Materials Science and Optoelectronics Engineering, University of Chinese Academy of Sciences, Beijing 100049, China.

^4^2020 X-Lab, Shanghai Institute of Microsystem and Information Technology, Chinese Academy of Sciences, Shanghai 200050, China.

^5^School of Graduate Study, University of Chinese Academy of Sciences, Beijing 100049, China.

*Corresponding authors. E-mail addresses: min.tu@mail.sim.ac.cn (M. Tu); xia.liu@bit.edu.cn (X. Liu).

**Contents of supporting information**

**Fig. S1** SEM images of SnO_2_ films deposited at different printing speeds 1

**Fig. S2** SnO_2_ film thickness measured by profilometry 2

**Fig. S3** Responses of SnO_2_ sensors at different operating temperatures. 3

**Fig. S4** SnO_2_ sensor responses toward four gases 4

**Fig. S5.** Stability and repeatability investigation of SnO_2_ gas sensor………………... 5

**Fig. S6** Simultaneous spark ablation of Sn and Au for tunable Au/SnO_2_ composite deposition 6

**Fig. S7** Responses of Au/SnO_2_ sensors under different operating temperatures 7

**Fig. S8** Au/SnO_2_ sensor responses toward four gases 8

**Fig. S9** Calibration and baseline noise analysis of the 7.8 wt% Au/SnO_2_ sensor used for LOD determination. 9

**Fig. S10** Dual sensitization mechanisms of Au/SnO_2_ gas sensors. 10

**Fig. S11** Responses of ZnO sensors at different operating temperatures. 11

**Fig. S12** Responses of NiO sensors at different operating temperatures 12

**Fig. S13** ZnO sensor responses toward four gases 13

**Fig. S14** NiO sensor responses toward four gases 14

**Fig. S15** Humidity-dependent responses of ZnO and NiO sensors to H_2_S 15

**Table S1.** Spark voltages and currents used to deposit Au/SnO_2_ 16

**Table S2.** Gas sensing performance of sensors fabricated via spark ablation in comparison to other methods 17

Table S3. Gas classification performance of ML-assisted sensor arrays in this work compared with recent reports 18

**
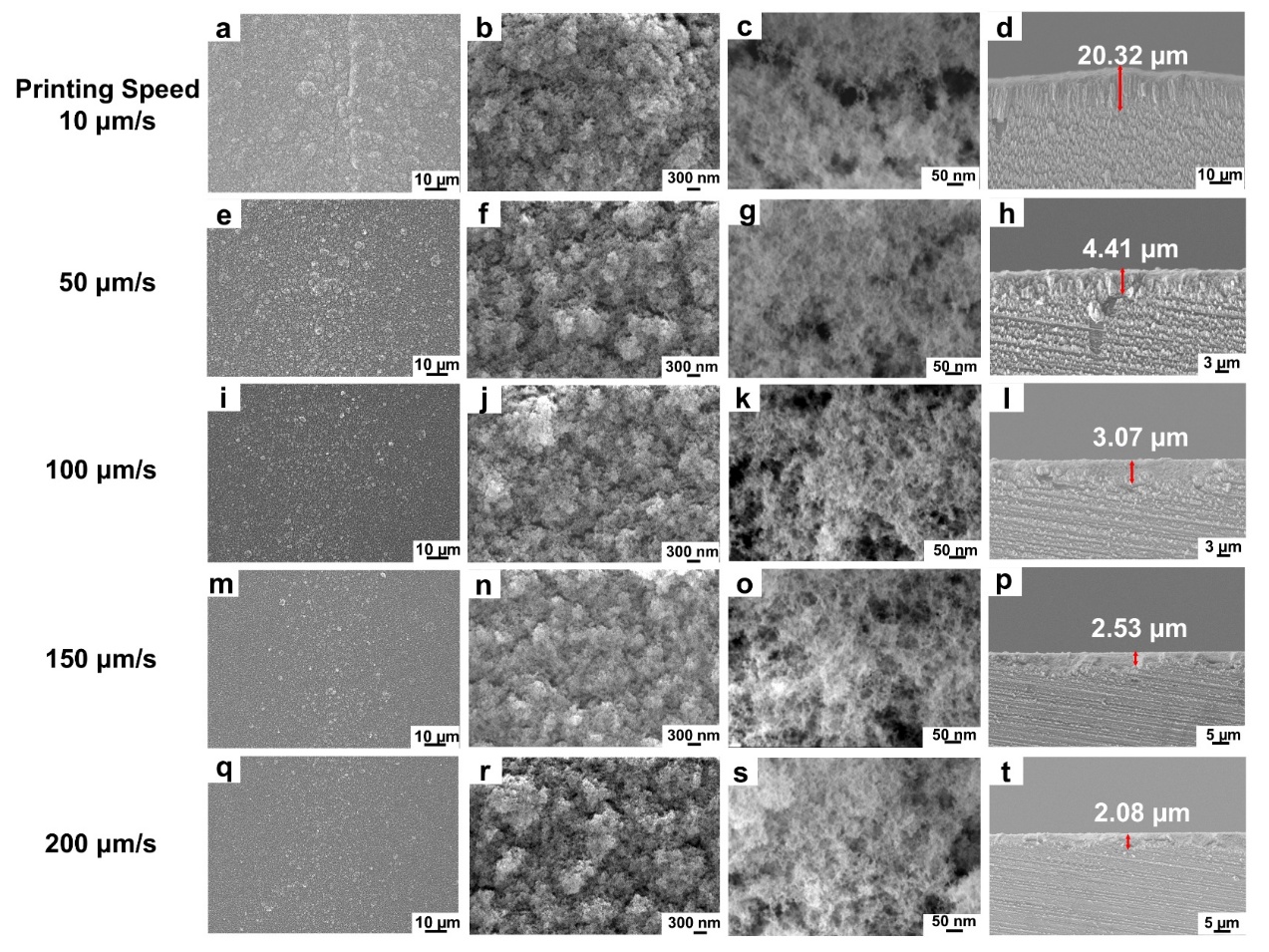
**

Figure S1. SEM images of SnO_2_ films printed at different speeds: 10 μm/s (a-d), 50 μm/s (e-h), 100 μm/s (i-l), 150 μm/s (m-p), and 200 μm/s (q-t). Columns 1-3 are top-view SEM images at increasing magnifications showing porous morphology across speeds; Column 4 are cross-sectional SEM images showing thickness reduction with increasing speed (d: 20.32 μm; h: 4.41 μm; l: 3.07 μm; p: 2.53 μm; t: 2.08 μm).


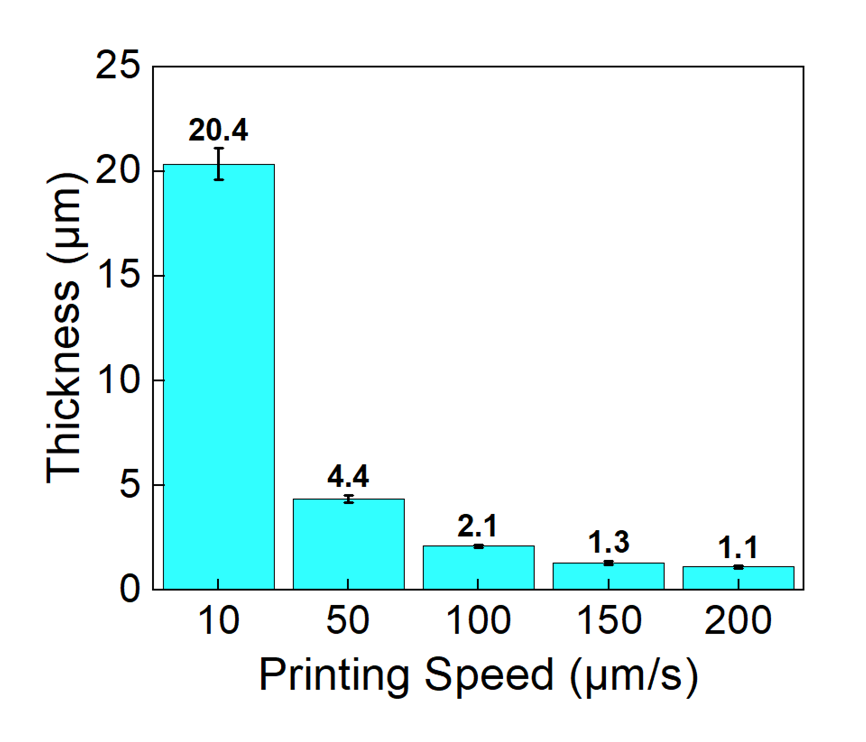


Figure S2. SnO_2_ film thickness measured by profilometry at different printing speeds (10-200 μm/s). The thickness decreases with increasing speed, consistent with the cross-sectional SEM observations.


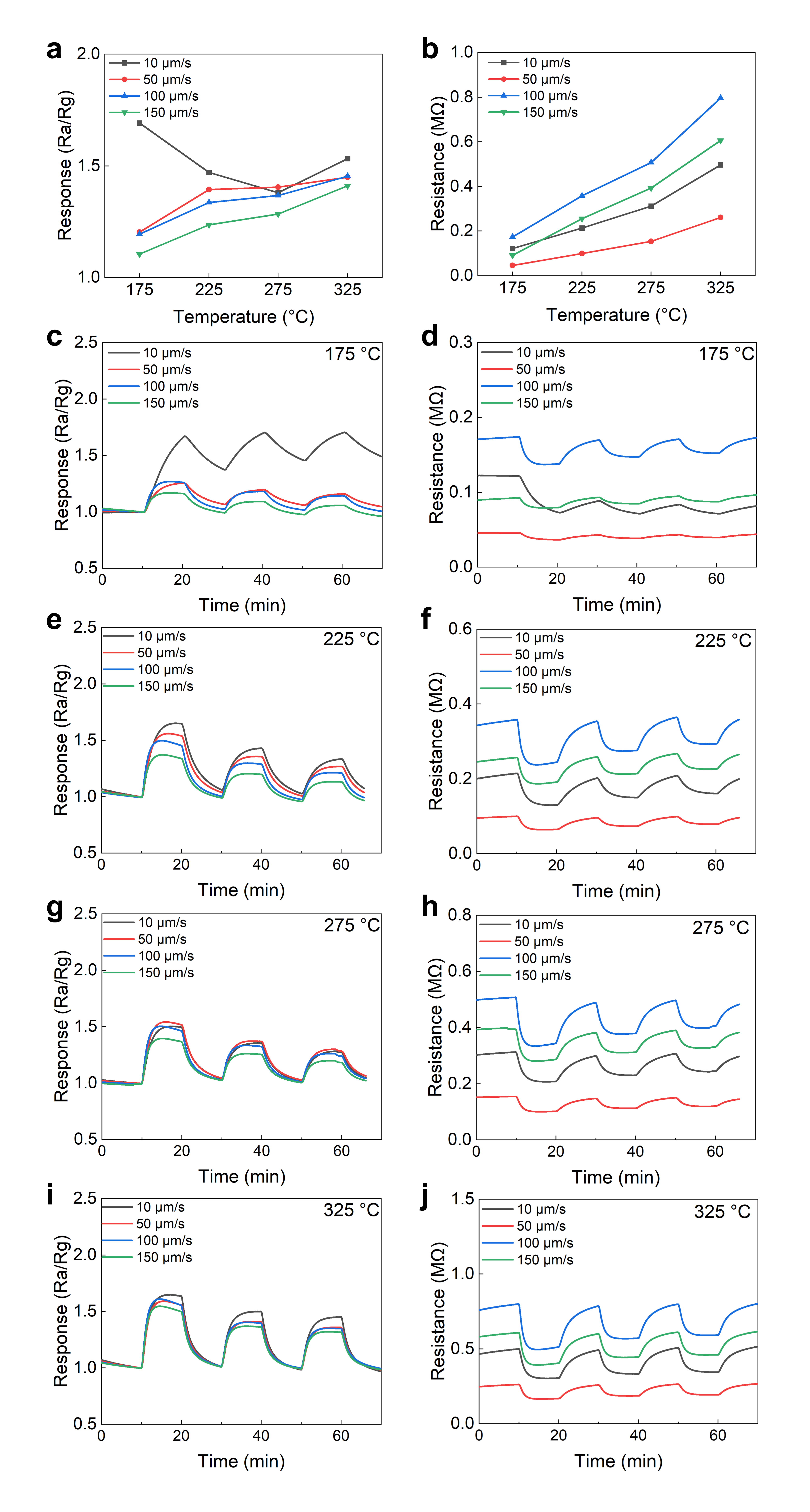


Figure S3. Dynamic responses of SnO_2_ sensors deposited at different printing speeds (10-150 μm/s) toward 2 ppm H_2_ at various operating temperatures. (a, b) Gas response and baseline resistance as a function of temperature (175-325 °C). (c, d) Dynamic response and resistance curves at 175 °C, (e, f) at 225 °C, (g, h) at 275 °C, and (i, j) at 325 °C. Each test includes three repeated exposure cycles. Based on these results, 325 °C was selected as the optimal operating temperature because it provided the highest response across cycles.


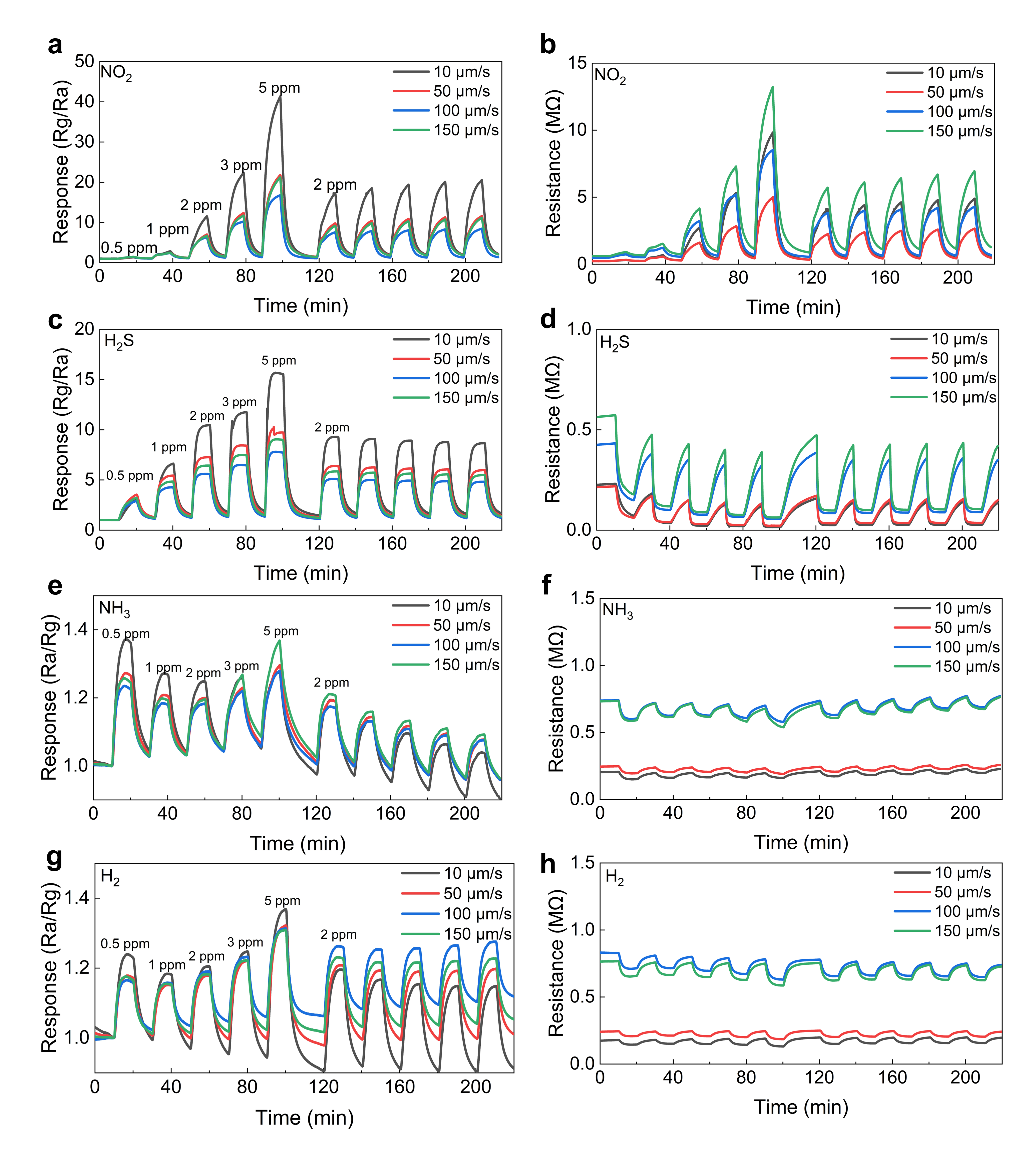


Figure S4. Gas responses and resistance characteristics of SnO_2_ sensors printed at different speeds (10-150 μm/s) toward (a, b) NO_2_, (c, d) H_2_S, (e, f) NH_3_, and (g, h) H_2_ at 325 °C. The tests were conducted with concentrations from 0.5 to 5 ppm, followed by five repeated cycles at 2 ppm. The SnO_2_ sensor exhibits reversible dynamic responses to gas concentrations, and the response is highly consistent across repeated cycles.

**Figure S5.** Stability and repeatability investigation of SnO_2_ gas sensor. (a) Dynamic response of a one-month-aged SnO_2_ -based sensor to 2 ppm, with 5 repetitions per phase over 3 phases during 8 hours. (b) Response of the SnO_2_ -based sensor to 2 ppm NO_2_ before (red) and after one month of aging (blue, data derived from the three phases of response repeatability tests to 2 ppm NO_2_). All tests were conducted at 325 °C

**
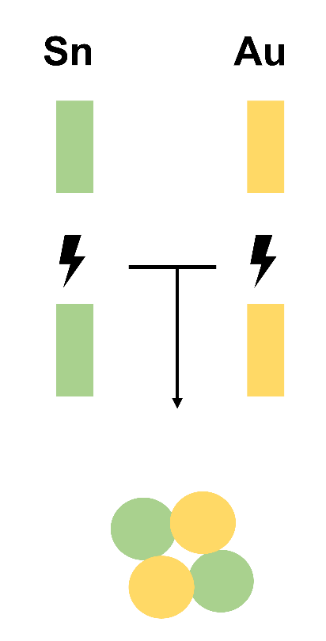
**

Figure S6. Schematic illustration of simultaneous spark ablation of Sn and Au electrodes, enabling the direct deposition of SnO_2_ with Au loadings.


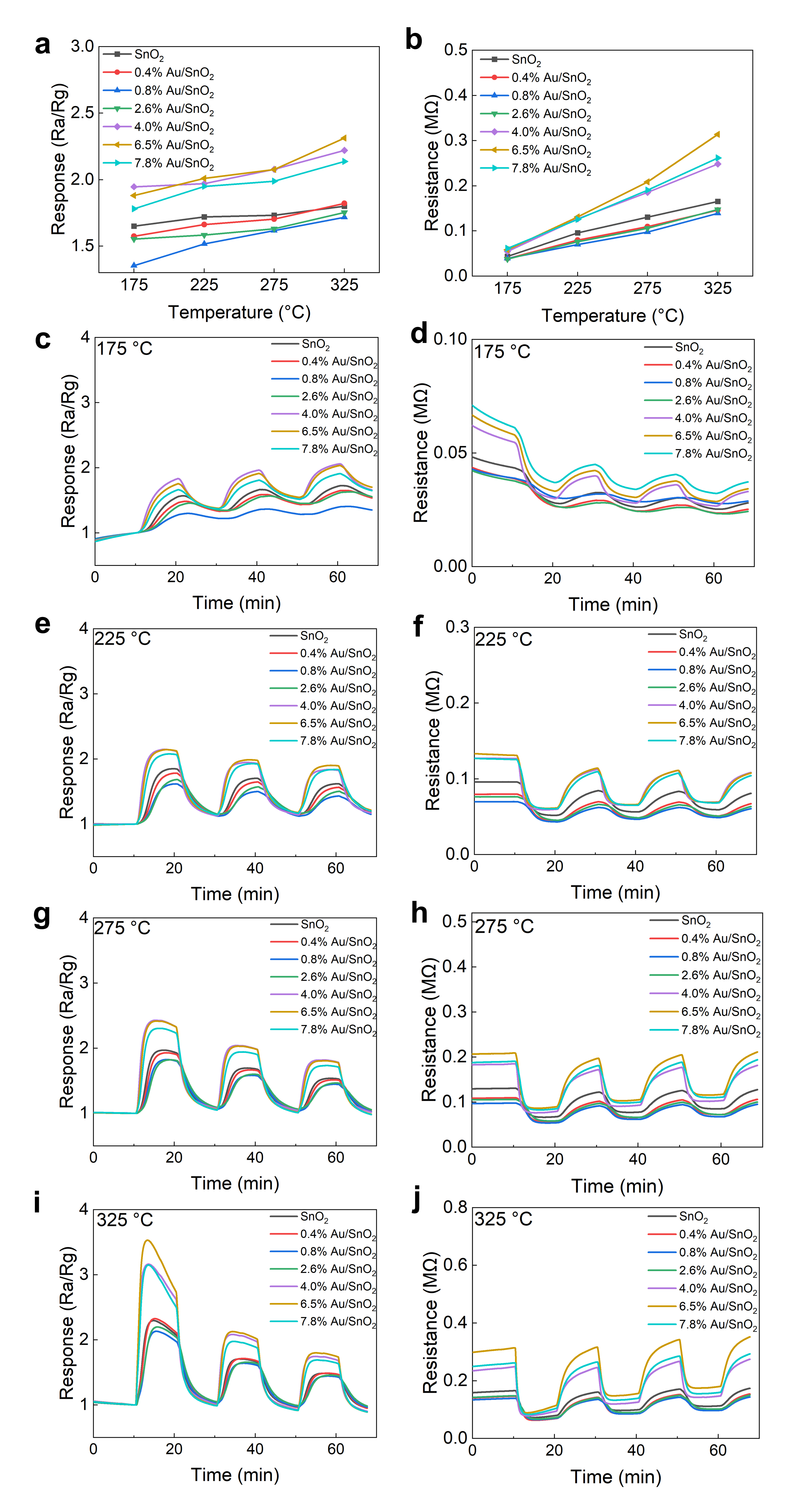


Figure S7. Dynamic responses of Au-loaded SnO_2_ sensors with different Au loadings (0-7.8 wt%) toward 2 ppm H_2_ at various operating temperatures. (a, b) Gas response and baseline resistance as a function of temperature (175-325 °C). (c, d) Dynamic response and resistance curves at 175 °C, (e, f) at 225 °C, (g, h) at 275 °C, and (i, j) at 325 °C. Each test includes three repeated exposure cycles. Based on these results, 275 °C was selected as the optimal operating temperature because it provided the higher response and better reproducibility across cycles.


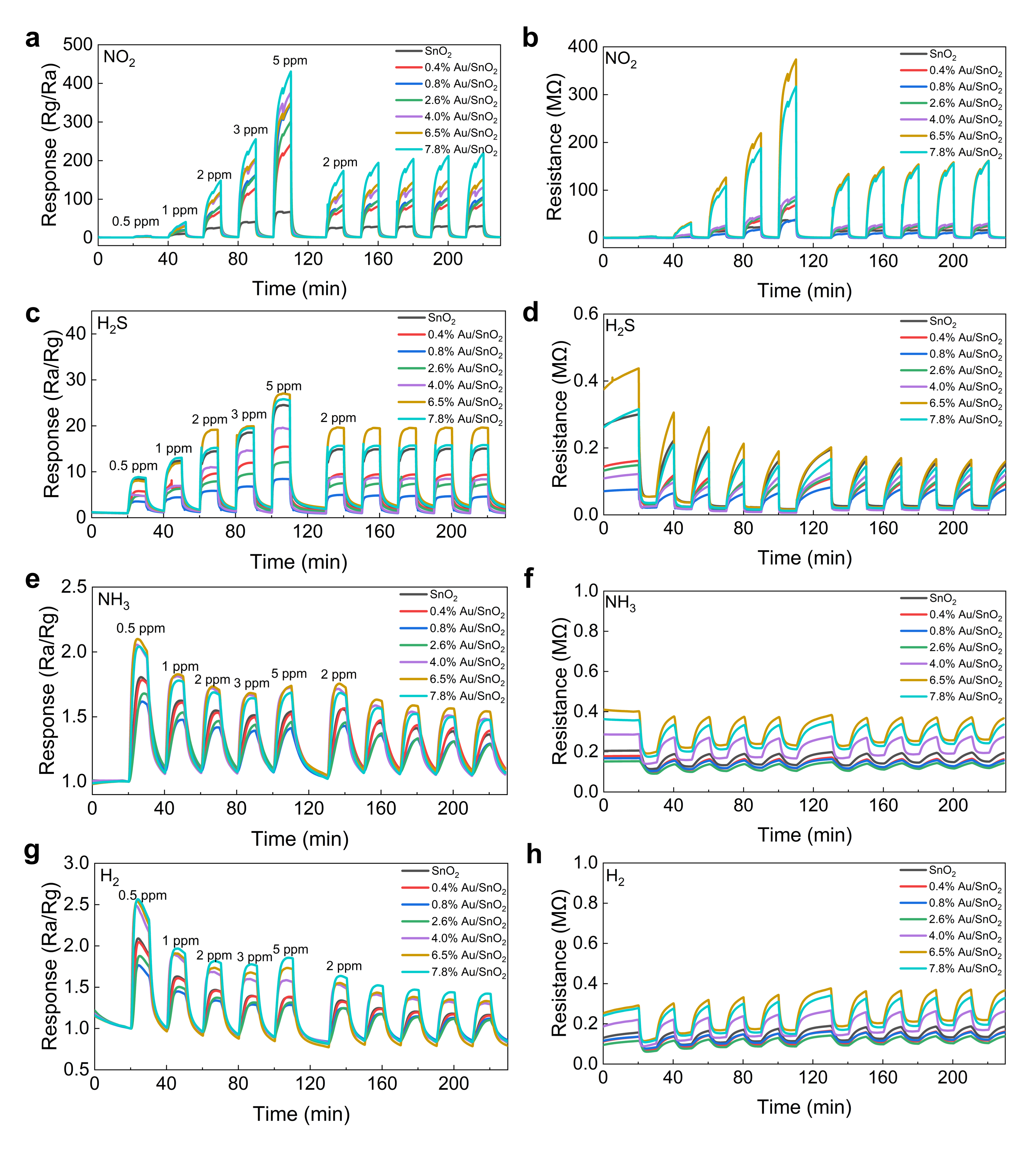


Figure S8. Dynamic gas responses and resistance changes of Au-loaded SnO_2_ sensors with different loadings (0-7.8 wt%) at 275 °C toward NO_2_ (a, b), H_2_S (c, d), NH_3_ (e, f), and H_2_ (g, h). Each test covers concentrations ranging from 0.5 to 5 ppm with five repeated cycles at 2 ppm. The Au/SnO_2_ sensors exhibit reversible dynamic responses to gas concentrations, with highly consistent performance across cycles.


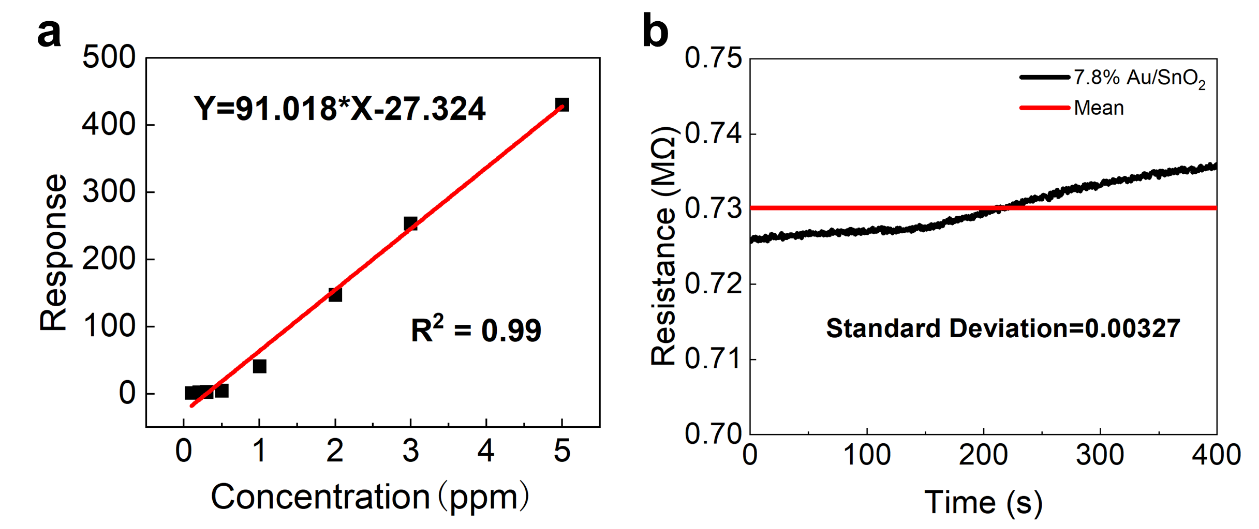


Figure S9. Calibration and baseline noise analysis of the 7.8 wt% Au/SnO_2_ sensor used for LOD determination. (a) Linear calibration curve in the low-concentration region, where the slope of the fit is S = 91.018 (R² = 0.99). (b) Baseline response of the same sensor in dry air over 400 s, giving a standard deviation of Sᵧ = 0.00327.

The relevant surface reactions include:^[1,2]^

O_2​_(ads) + e^−^→ O^−^ (ads) (1)

NO_2​_​(ads) + e^−^→ NO_2​_^−^​(ads) (2)

2NO_2​_^−^​ + O^−^→ 2NO_3_^−^​ (3)​


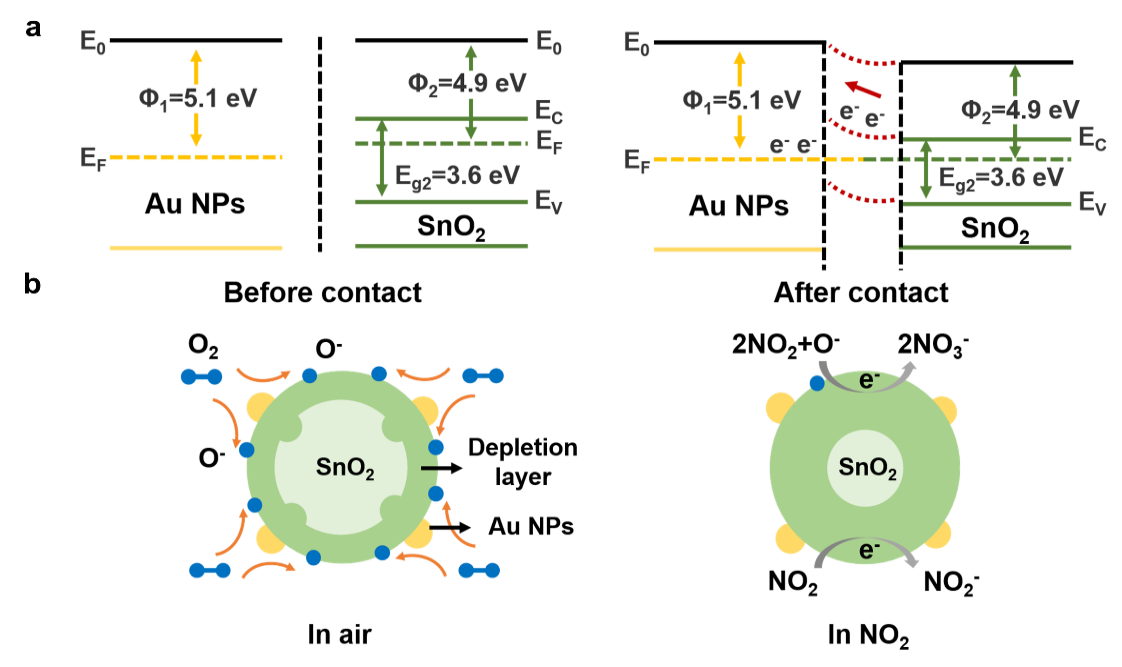


Figure S10. Mechanisms and sensing performance of Au-loaded SnO_2_ gas sensors. (a) Electronic sensitization: Au nanoparticles modulate carrier concentration by forming a Schottky barrier at the Au-SnO_2_ interface. (b) Chemical sensitization: Au nanoparticles catalyze oxygen activation and NO_2_ adsorption, enhancing surface reactions and improving sensing performance.


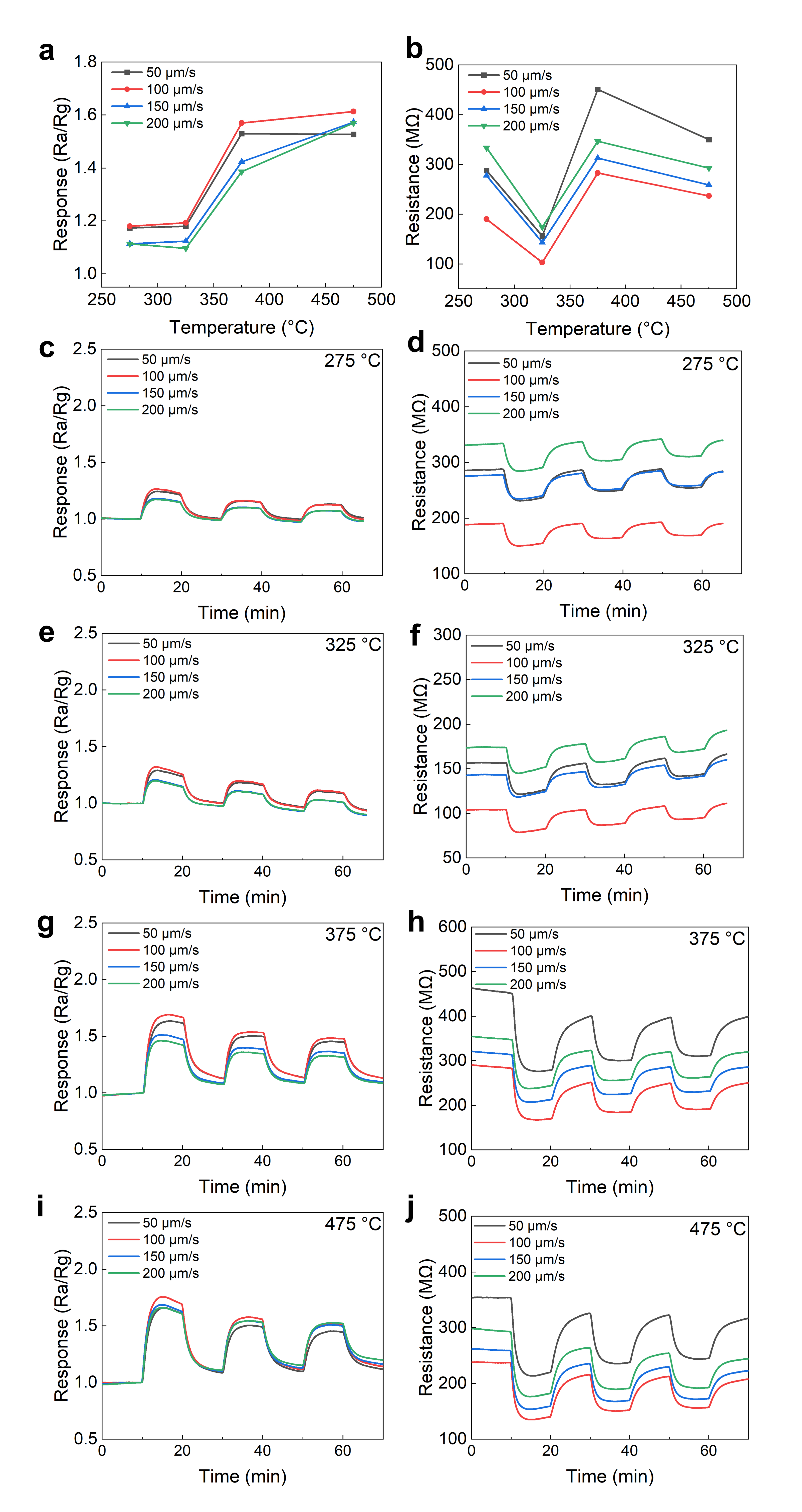


Figure S11. Dynamic responses of ZnO sensors deposited at different printing speeds (50-200 μm/s) toward 2 ppm H₂ at various operating temperatures. (a, b) Gas response and baseline resistance as a function of temperature (275-475 °C). (c, d) Dynamic response and resistance curves at 275 °C, (e, f) at 325 °C, (g, h) at 375 °C, and (i, j) at 475 °C. Each test includes three repeated exposure cycles. Based on these results, 475 °C was selected as the optimal operating temperature because it provided the highest response and lower baseline resistance across cycles.


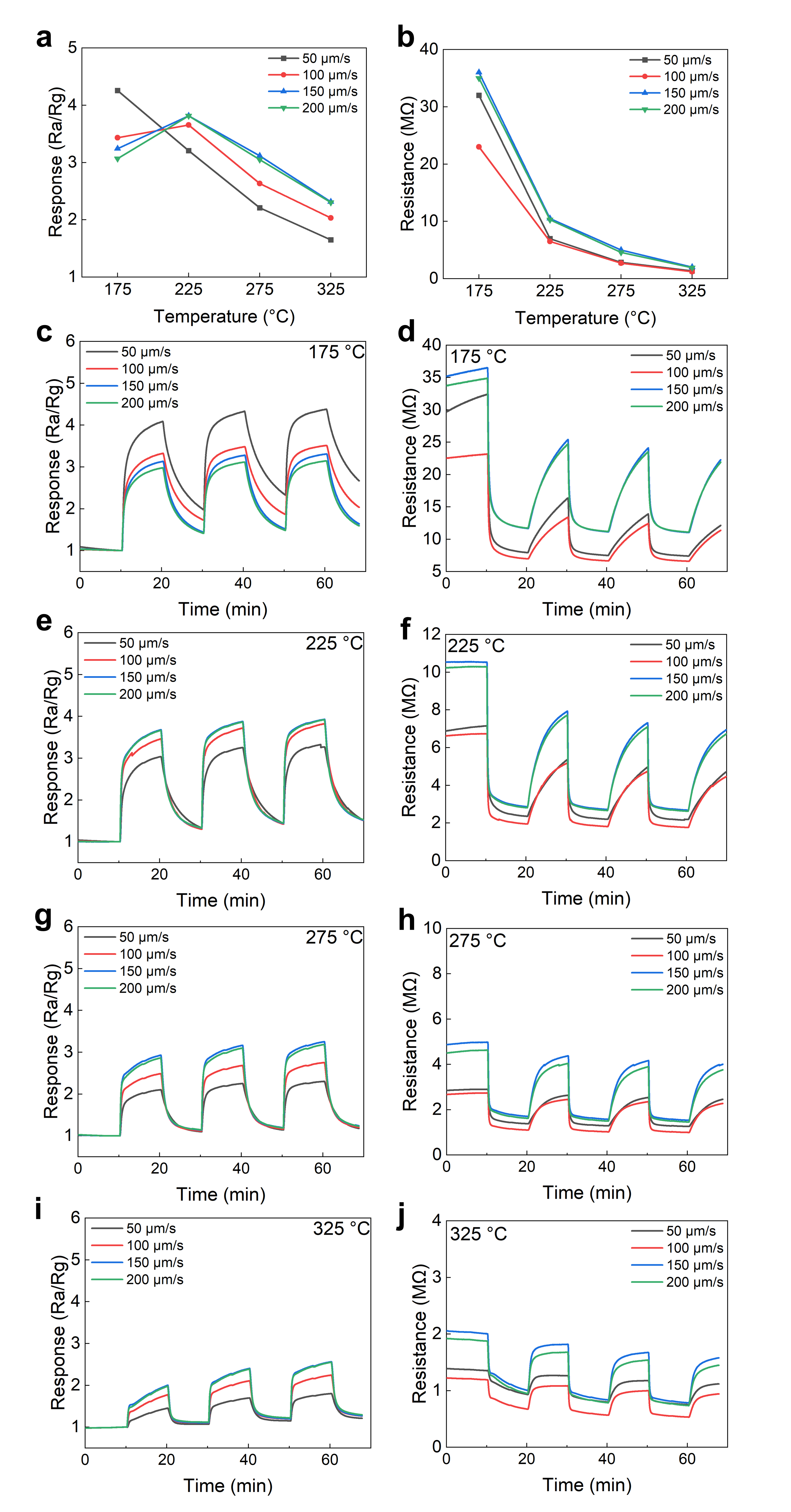


Figure S12. Dynamic responses of NiO sensors deposited at different printing speeds (50-200 μm/s) toward 5 ppm NO_2_ at various operating temperatures. (a-b) Gas response and baseline resistance as a function of temperature (175-325 °C). (c-d) Dynamic response and resistance curves at 175 °C, (e-f) at 225 °C, (g-h) at 275 °C, and (i-j) at 325 °C. Each test includes three repeated exposure cycles. Based on these results, 225 °C was selected as the optimal operating temperature because it provided the highest response and better reproducibility across cycles.


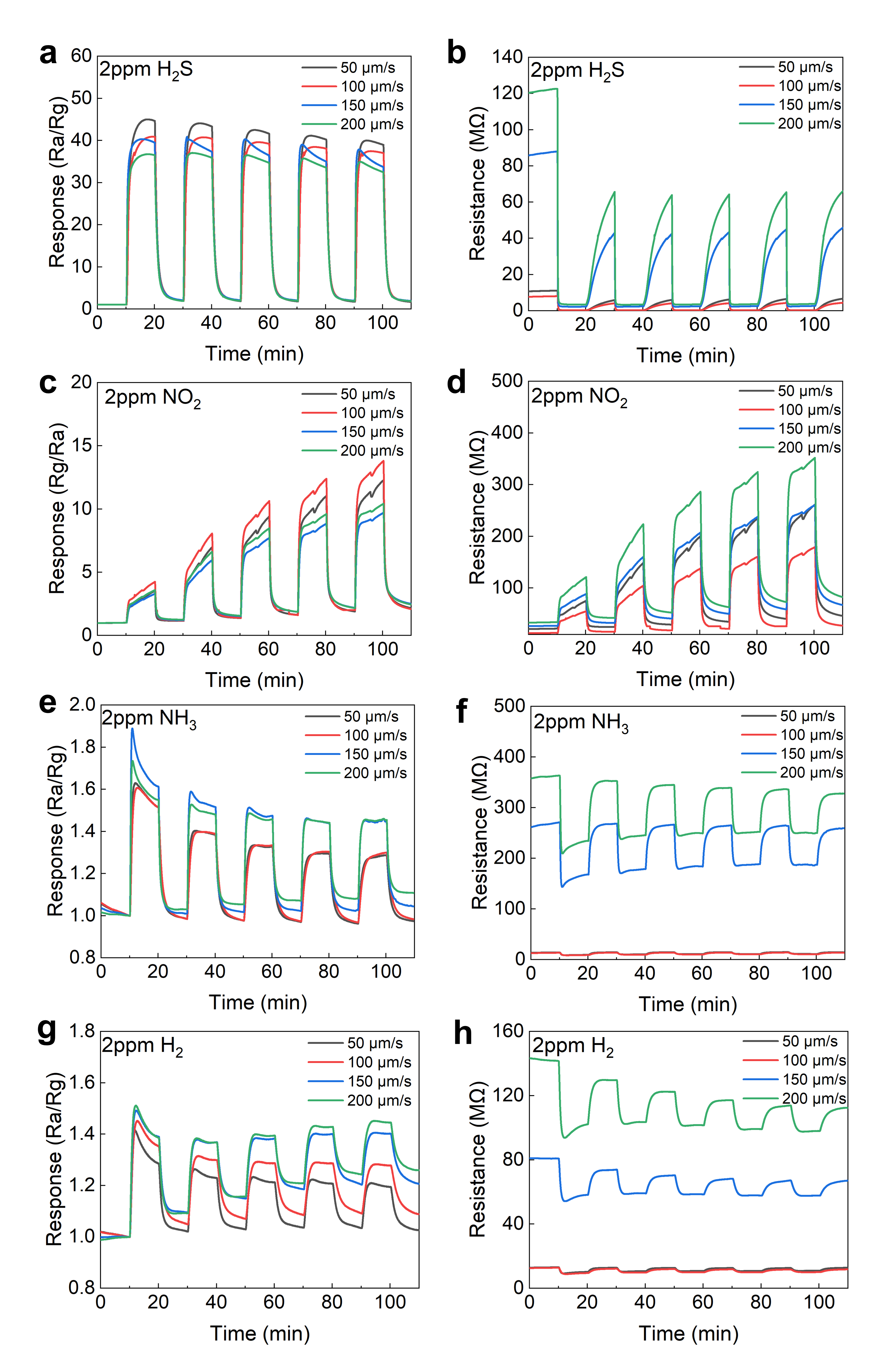


Figure S13. Gas response and resistance of ZnO sensors deposited at different printing speeds (50-200 μm/s) toward H_2_S (a-b), NO_2_ (c-d), NH_3_ (e-f), and H_2_ (g-h) at 475 °C. Each gas test was conducted at a fixed concentration of 2 ppm with five repeated exposure cycles, showing good consistent performance across cycles.


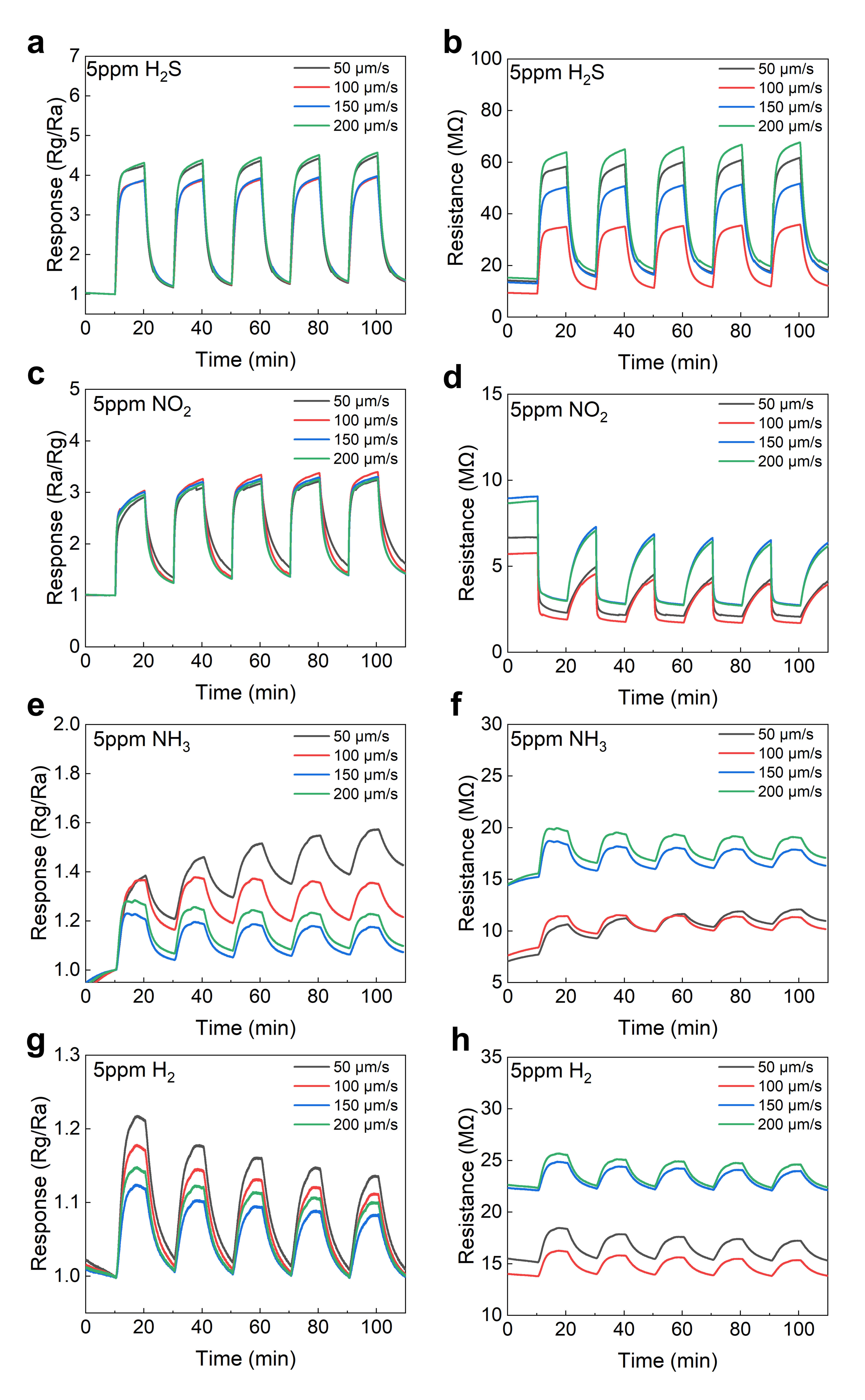


Figure S14. Gas response and resistance of NiO sensors deposited at different printing speeds (50-200 μm/s) toward H_2_S (a-b), NO_2_ (c-d), NH_3_ (e-f), and H_2_ (g-h) at 225 °C. Each gas test was conducted at a fixed concentration of 5 ppm with five repeated exposure cycles, showing good consistent performance across cycles.

aa
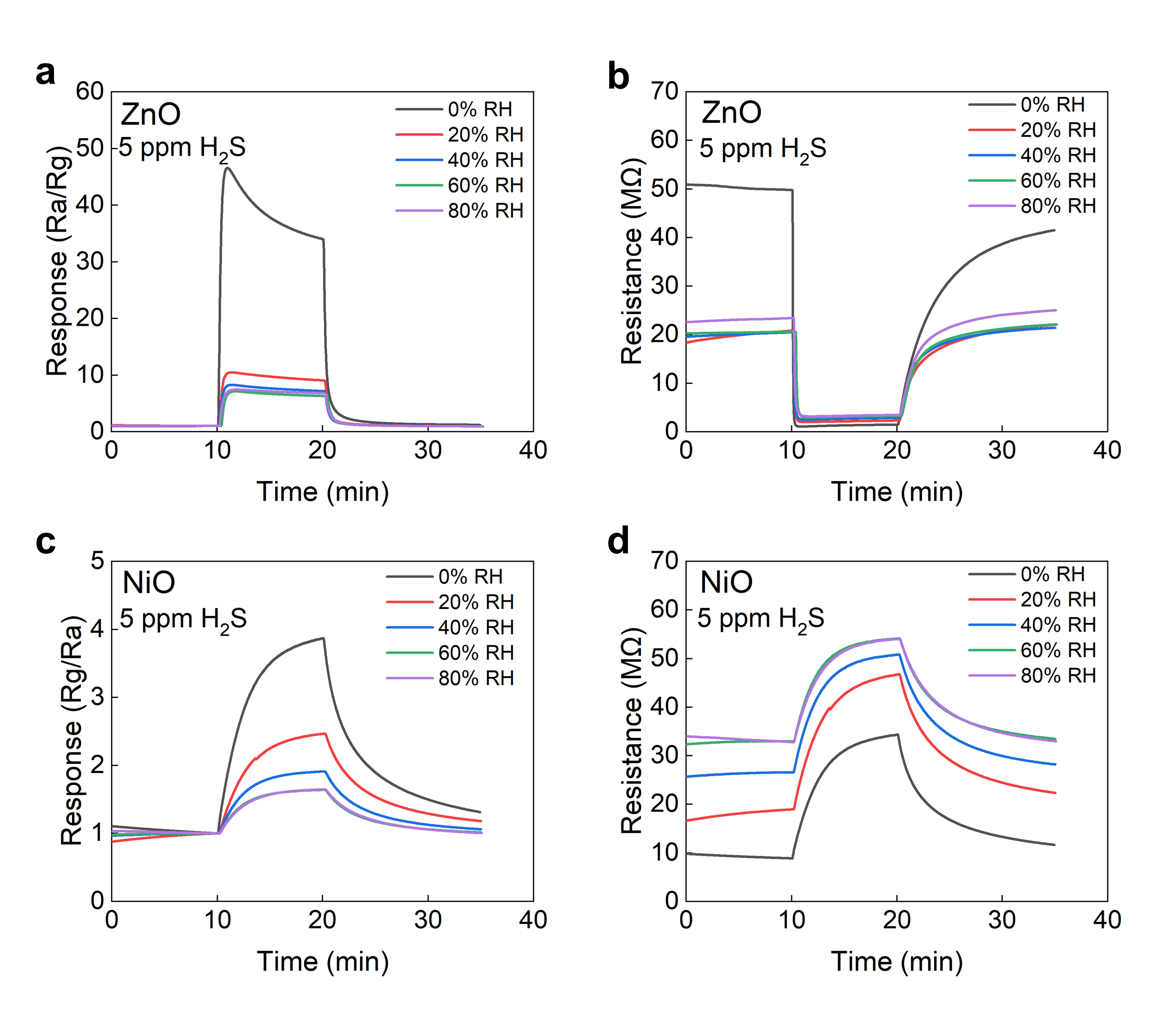


Figure S15. Response and resistance curves of ZnO (a-b) and NiO (c-d) sensors toward 5 ppm H₂S under different relative humidity (RH) levels (0-80%). Increasing RH reduced the response due to competitive adsorption of water molecules, yet both sensors maintained stable baselines and reproducible signal profiles, demonstrating reliable operation under realistic environmental conditions.

**Supplementary Table**

Table S1. Spark voltages and currents used to deposit Au/SnO_2_ with different Au amount.

| **Au wt%^a)^** | **Au Voltage (kV)** | **Au Current (mA)** | **Au Output (mg/h)** | **SnO_2_ Voltage (kV)** | **SnO_2_ Current (mA)** | **SnO_2_ Output (mg/h)** |
| --- | --- | --- | --- | --- | --- | --- |
| **0.4%** | 0.2 | 0.1 | 0.01 | 1.3 | 8 | 2.59 |
| **0.8%** | 0.2 | 0.2 | 0.02 | 1.3 | 8 | 2.59 |
| **2.6%** | 0.2 | 0.7 | 0.07 | 1.3 | 8 | 2.59 |
| **4.0%** | 0.2 | 1.1 | 0.11 | 1.3 | 8 | 2.59 |
| **6.5%** | 0.4 | 0.9 | 0.18 | 1.3 | 8 | 2.59 |
| **7.8%** | 0.4 | 1.1 | 0.22 | 1.3 | 8 | 2.59 |

**^a)^** The Au amount was calculated as the weight percentage (wt%) using the actual mass of Au divided by the total mass of Au and SnO_2_, i.e., Au wt% = (m(Au) / (m(Au) + m(SnO_2_))) × 100%. The masses of Au and SnO_2_ were derived from the theoretical yields estimated based on the corresponding discharge voltages and currents.

Table S2. Gas sensing performance of sensors fabricated via spark ablation in comparison to other methods.

| **Material** | **Synthesis method** | **Target gas** | **Concentration (ppm)** | **Response** | **LOD**  **(ppb)** | **Ref.** |
| --- | --- | --- | --- | --- | --- | --- |
| SnO_2_ | - | H_2_S | 50 | 3 | 1000 | Commercial |
| SnO_2_ | MOF-derived | NO_2_ | 1 | 64 | 10 | [3] |
| SnO_2_ | hydrothermal | NO_2_ | 3 | 25.4 | 100 | [4] |
| SnO_2_ | Hydrothermal | formaldehyde | 10 | 15 | 59 | [5] |
| **SnO_2_** | **spark ablation** | **NO_2_** | **2** | **16.28** | **1** | **this work** |
| Au/SnO_2_ | sol-gel | H_2_S | 0.5 | 278 | 0.288 | [6] |
| Au/SnO_2_ | hydrothermal synthesis | NO_2_ | 1 | 1190 | 2 | [7] |
| Au/SnO_2_ | ALD | toluene | 1 | 5.33 | 0.75 | [8] |
| **Au/SnO_2_** | **spark ablation** | **NO_2_** | **2** | **191.55** | **0.11** | **this work** |
| Cu_2_O/ZnO | Confined 2D Electrodeposition | H_2_S | 0.5 | 1660 | 10 | [9] |
| ZnO | Biotemplate-assisted | H_2_S | 10 | 85.04 | 10 | [10] |
| ZnO | aqueous ink | NO_2_ | 100 | 33400 | 2 | [11] |
| **ZnO** | **spark ablation** | **H_2_S** | **2** | **27.88** | **0.17** | **this work** |
| CuO/NiO | hydrothermal synthesis | H_2_S | 5 | 36.9 | 0.5 | [12] |
| NiO | wet-chemical | Acetone | 0.2 | 107.7 | 0.8 | [13] |
| NiO | [inkjet printing](https://www.sciencedirect.com/topics/engineering/inkjet-printing) | H_2_ | 1000 | 32.1 | 2500 | [14] |
| **NiO** | **spark ablation** | **H_2_S** | **5** | **4.28** | **2.5** | **this work** |

Table S3. Gas classification performance of ML-assisted sensor arrays in this work compared with recent reports.

| **Target Gases** | **ML Algorithm** | **Accuracy (%)** | **Reference** |
| --- | --- | --- | --- |
| VOCs | Extra Trees | 94.8 | [15] |
| NH_3_, H_2,_ CO | KNN | 100 | [16] |
| NO_2_, SO_2_, NO | SNN | 98.2 | [17] |
| VOCs | RF | 99 | [18] |
| **NO_2_, H_2_S, NH_3_, H_2_** | **RF** | **>99.2** | **this work** |

**References**

Maiti, A., Rodriguez, J. A., Law, M., Kung, P. & McKinney, J. R. SnO_2_ nanoribbons as NO₂ sensors: insights from first principles calculations. *Nano Lett.* **3**, 1025–1028 (2003).

Yuan, C. et al. Modeling interfacial interaction between gas molecules and semiconductor metal oxides: a new view angle on gas sensing. *Adv. Sci*. **9**, 2203594 (2022).

Li, X. et al. Trace detection of nitrogen dioxide via porous tin dioxide nanopods with high specific surface area and enhanced charge transfer. *ACS Sens.* **10**, 4383–4390 (2025).

Li, J. et al. Fast detection of NO_2_ by porous SnO_2_ nanotoast sensor at low temperature. *J. Hazard. Mater.* **419**, 126414 (2021).

Wang, Y.-N. et al. Synergistic effect of high-energy (211) facets and (101) twin defects of SnO_2_ nanowires for ultrasensitive ppb-level formaldehyde monitoring. *Adv. Funct. Mater.* **n/a**, e12541 (2025).

Deb, M., Lu, C.-J. & Zan, H.-W. Achieving room-temperature ppb-level H_2_S detection in a Au–SnO_2_ sensor with low voltage enhancement effect. *ACS Sens.* **9**, 4568–4577 (2024).

Tofighi, G. et al. Microfluidically synthesized Au, Pd and AuPd nanoparticles supported on SnO_2_ for gas sensing applications. *Sens. Actuators B Chem.* **292**, 48–56 (2019).

Yan, J., Kang, Y., Fang, W., Zhu, B. & Song, Z. Tuning gas sensing properties through metal-nanocluster functionalization of 3D SnO_2_ nanotube arrays for selective gas detection. *ACS Sens.* **10**, 6084–6094 (2025).

Chen, H., Lv, L., Xue, K., Zhang, P. & Du, L. Oral exhalation H₂S sensor based on Cu_2_O/ZnO heterostructures. *ACS Sens.* **10**, 2579–2588 (2025).

Na, H.-B., Zhang, X.-F., Deng, Z.-P., Xu, Y.-M. & Huo, L.-H. Large-scale synthesis of hierarchically porous ZnO hollow tubule for fast response to ppb-level H_2_S gas. *ACS Appl. Mater. Interfaces* **11**, 11627–11635 (2019).

Li, G. et al. Adjustment of oxygen vacancy states in ZnO and its application in ppb-level NO_2_ gas sensor. *Sci. Bull*. **65**, 1650–1658 (2020).

Sui, L., Yu, T., Zhao, D., Cheng, X. & Zhang, X. In situ deposited hierarchical CuO/NiO nanowall arrays film sensor with enhanced gas sensing performance to H₂S. *J. Hazard. Mater.* **385**, 121570 (2020).

Li, C., Choi, P. G., Kim, K. & Masuda, Y. High performance acetone gas sensor based on ultrathin porous NiO nanosheet. *Sens. Actuators B Chem.* **367**, 132143 (2022).

Yan, W., Luo, W. & Li, M. NiO nanoparticles-based gas sensors: a novel pulse-driven approach for enhanced and efficient hydrogen detection. Int. *J. Hydrogen Energy* **85**, 481–488 (2024).

N. Vadera, S. Dhanekar, Classification and Prediction of VOCs Using an IoT-Enabled Electronic Nose System-Based Lab Prototype for Breath Sensing Applications, *ACS Sens*. **10,** 439–447 (2025).

W. Luo, F. Dai, Y. Liu, X. Wang, M. Li, Pulse-driven MEMS gas sensor combined with machine learning for selective gas identification, *Microsyst Nanoeng* **11,** 72 (2025).

D.-B. Moon, A. Bag, H.H. Chouhdry, S.J. Hong, N.-E. Lee, Selective Identification of Hazardous Gases Using Flexible, Room-Temperature Operable Sensor Array Based on Reduced Graphene Oxide and Metal Oxide Nanoparticles via Machine Learning, *ACS Sens*. **9,** 6071−6081 (2024).

B. Wang, J. Zhang, T. Wang, W. Li, Q. Lu, H. Sun, L. Huang, X. Liang, F. Liu, F. Liu, P. Sun, G. Lu, Machine Learning-Assisted Volatile Organic Compound Gas Classification Based on Polarized Mixed-Potential Gas Sensors, *ACS Appl. Mater. Interfaces* **15**, 6047–6057 (2023).
